# Integrating Cycled Enzymatic DNA Amplification and Surface-Enhanced Raman Scattering for Sensitive Detection of Circulating Tumor DNA

**DOI:** 10.3389/fmolb.2021.676065

**Published:** 2021-05-04

**Authors:** Xinxing Miao, Qianqian Fang, Xiang Xiao, Sidi Liu, Renfei Wu, Jun Yan, Baoqing Nie, Jian Liu

**Affiliations:** ^1^Institute of Functional Nano and Soft Materials, Jiangsu Key Laboratory for Carbon-Based Functional Materials and Devices, Soochow University, Suzhou, China; ^2^School of Electronic and Information Engineering, Soochow University, Suzhou, China

**Keywords:** surface-enhanced raman scattering (SERS), enzymatic amplification, circulating tumor DNA (ctDNA), DNA nanotechnology, biomarker

## Abstract

Circulating tumor DNA (ctDNA) represents an emerging biomarker of liquid biopsies for the development of precision cancer diagnostics and therapeutics. However, sensitive detection of ctDNA remains challenging, due to their short half-life and low concentrations in blood samples. In this study, we report a new method to address this challenge by integrating cycled enzymatic DNA amplification technique and Au nanoparticle@silicon-assisted surface-enhanced Raman scattering (SERS) technique. We have demonstrated a reproducible identification of a single-base-mutated ctDNA sequence of diffuse intrinsic pontine gliomas (DIPGs), with the limit of detection (LOD) as low as 9.1 fM in the spiked blood samples. This approach can be used to analyze trace amounts of ctDNA in translational medicine for early diagnosis, therapeutic effect monitoring, and prognosis of patients with cancer.

## Introduction

Circulating tumor DNA (ctDNA) carries genetic information, such as point mutations, methylation, and copy number variations of sequences, of the tumor cells in patients with cancer, thus simultaneously serving as a diagnostic as well as a prognostic cancer biomarker based on liquid biopsies (Dawson et al., 2013; Weiss et al., 2013; Alix-Panabieres and Pantel, 2016; Wan et al., 2017; Reinert et al., 2018). Previous studies suggest that ctDNA is mainly derived from the processes of cell apoptosis, necrosis, and secretion (Diaz and Bardelli, 2014). The typical concentration of ctDNA in the clinical samples is in the range of 0.01−0.1 ng ml^–1^, accounting for only a small fraction (nearly 1%) of the total cell-free DNA (cfDNA; Diehl et al., 2008; Wan et al., 2017). In addition, ctDNA features a much shorter half-life (<2 h) compared with other protein biomarkers, which usually remain for several weeks (Dawson et al., 2013; Bettegowda et al., 2014), and susceptibility to the variants in the liquid biopsy handling procedure. Technically, it is challenging to distinguish ctDNA with the fewest point mutations from wild-type DNA (W) and quantify the concentrations of ctDNA from the complicated background interference of biological samples.

The most widely used technologies for ctDNA detection include varieties of methods based on PCR, such as digital PCR (dPCR; Vogelstein and Kinzler, 1999; Taly et al., 2013); amplification refractory mutation system (ARMS; Newton et al., 1989; Li et al., 2006); beads, emulsion, amplification, magnetics (BEAMing; Li et al., 2006); next-generation gene sequencing (NGS) technology (Forshew et al., 2012; Lai et al., 2018); and Sanger sequencing (Janne et al., 2006). However, there are concerns of generating false-positive results due to the ultra-high amplification capacity enabled by polymerases (Kitchin et al., 1990; Mathai and Adhikari, 2013). Many researchers have been trying to develop non-PCR-based techniques to detect ctDNA, including electrochemistry (Drummond et al., 2003; Yang et al., 2016), colorimetry (Li et al., 2017), and fluorescence (Li et al., 2016). Among them, surface-enhanced Raman scattering (SERS) spectroscopy has been considered an effective solution, due to its several advantages, such as ultra-high sensitivity, identification with characteristic fingerprint peaks, and the compatibility of both solid-form and liquid-form samples (Liang et al., 2019; Szekeres and Kneipp, 2019; Chen et al., 2020; Du et al., 2020; Fan et al., 2020; Langer et al., 2020; Li et al., 2020). Lin et al. (2019) reported sensitive detection of ctDNA for a good diagnostic sensitivity of 83.3% and a specificity of 82.5% in distinguishing patients with nasopharyngeal cancer from normal control groups by using the SERS substrate of Ag nanoparticles. Wee et al. designed a new laser wrapped graphene–Ag array to sensitively detect the methylated DNA by using SERS technology and demonstrated a limit of detection (LOD) of as low as 0.2 pg μL^–1^ (Wee et al., 2016). There is still a great need to improve the LOD for the translational research of ctDNA.

Herein, we report a new method for sensitive detection of the sequence of ctDNA (H3.3 mutation) in diffuse intrinsic pontine gliomas (DIPGs) by using a combination of cycled enzymatic DNA amplification and Au nanoparticle@silicon (Au NP@Si)-assisted SERS technology. DIPGs are high-grade glial tumors located in the pons of the brain and are the leading cause of fatal brain tumors in children. The traditional diagnostic methods of DIPGs heavily rely on *in vivo* imaging, which can become difficult for identification because of the deep location of the tumor sites in the brain. In this study, we have designed a SERS-tag-labeled probe DNA (P), which complements the H3.3 mutant DNA (T) to form a blunt end. The cleavage assisted by EXO III can generate a large amount of residual DNA in a cycled manner to greatly improve the LOD of the SERS technique. We have demonstrated an ultra-high sensitivity (with the LOD of 7.9 fM) and specificity (being able to distinguish a single-base mutation) using this strategy. This is a promising approach for the sensitive detection of nucleic acids as a translational tool of ctDNA research.

## Experimental Section

### Materials and Reagents

Magnesium chloride hexahydrate (MgCl_2_⋅6H_2_O), sodium chloride (NaCl), acetone, and phosphate buffer saline (PBS) were purchased from Sinopharm Chemical Reagent Co., Ltd. (China). Exonuclease III was provided by Thermo Fisher Scientific Co., Ltd. (United States). Tris borate EDTA (TBE) buffer (5×), tris EDTA (TE) buffer (1×), sodium dodecyl sulfate (SDS, 10%, w/v) buffer, loading dye buffer solutions (6×), agarose, and saline-sodium citrate (SSC, 20×) buffer solutions were obtained from Solarbio Science & Technology Co., Ltd. (China). GelRed Neuclic Acid Gel Stain was purchased from Biotium, Inc. (United States). DNA ladder was provided by Sigma-Aldrich Co., Ltd. (China). QIAamp DNA Blood Mini Kit was purchased from Qiagen Co., Ltd. (Germany). All chemicals in our experiments were of analytical grade and used without further purification. Aqueous solutions were prepared using deionized water (≥18 MΩ, Milli-Q, Millipore). The SERS substrates were provided by Nanova Biomaterials Inc. (United States). High-performance liquid chromatography (HPLC)-purified oligonucleotides were provided by Sangon Biotechnology Co., Ltd. (China).

### Instruments

The Raman microscope equipped with a He–Ne laser (633 nm, 20 mW) and a 100× objective (NA: 0.9) was used to detect the Au NP@Si substrates (HR800, Horiba Jobin Yvon, France). SERS data were collected under a 100× visible objective with a 633 nm laser radiation, with an acquisition time of 10 s and an accumulation count of one time. The scan of the wave number typically ranged from 1,000 to 1,800 cm^–1^. The Raman spectral data were analyzed by the LabSpec5.6 Software. For each sample, 50 random spots on the substrate were tested for SERS signal. All SERS spectra have been elaborated by removing the baseline, with an example shown in Supplementary Figure 1. The correlation of the major Raman bands to the chemical bonds of Cy5 is summarized in Table 1. A DNA thermostat hybridization oven (HL-2000 HybriLLinker, UVP, United States) was used for the incubation experiments involving DNA. The substrate surface was characterized by contact angle measurement instrument (DataPhysics OCA). The gel electrophoresis of various sample mixtures was performed on a horizontal electrophoresis tank instrument (VE-186), followed by image acquisition with a Gel Imaging System (Tanon-2500).

**TABLE 1:** List of the oligonucleotide sequences in the experiments.

**Oligonucleotides**	**Sequence (5′ → 3′)**
Probe DNA (P)	Cy5-AAAATGAGTGCGTAGTTAGGGTTAGATA AGGGCGCACTCATGCGA
Mutant DNA (T)	TCGCATGAGTGCGCCCTCTACT
Wildtype DNA (W)	TCGCAAGAGTGCGCCCTCTACT
Capture DNA (C)	GCACTCATTTTTAATTTAA
Residual DNA (R)	Cy5-AAAATGAGTGCGTAGTTAGGGTTAGATA

### Electrophoresis Experiments

The hybridization and the Exo III-assisted cleavage of different DNA samples were evaluated by agarose gel electrophoresis. Agarose (2%, w/w) was prepared in the buffer of 1× TBE and mixed with 3 μL of GelRed stain. Specifically, 10 μL of the DNA sample was mixed with 2 μL of 6× loading dye buffer solution. Different DNA samples were tested by gel electrophoresis under a voltage of 110 V for 25 min. The DNA sample mixtures included P, T, the hybridization of P and T (P + T), the mixture of the P, T, and Exo III (P + T + Exo III), and the residual DNA (R).

### ctDNA Detection on the Au NP@Si Substrate

The SERS signals of ctDNA testing experiments were harvested from the Au NP@Si substrate. In the first step, the Au NP@Si substrate was modified with the capture DNA (C) sequence. The substrate was precleaned with acetone, rinsed with DI water for three times, and dried in an oven at 200°C for 10 min. The pretreated substrate was incubated overnight with 10 μL of C (10 μM) and 400 μL of the PBS solution at 37°C for bioconjugation through Au–S bonds. The sodium chloride (1 M) solution was used in the aging step for immobilization of C (15 μL, three times with an interval of 20 min). The substrate was incubated overnight in the DNA thermostat hybridization oven at 37°C, followed by sequential washing with PBS and DI water three times. The reaction of cycled enzymatic DNA cleavage/amplification was carried out by mixing 10 μL of P (10 μM), 10 μL of ctDNA in the testing concentrations, 3 μL of Exo III (60U), and 17 μL of the enzyme-reaction buffer at 37°C for 2 h. Afterward, the mixture was incubated at 75°C for 10 min in order to inactivate the Exo III enzyme. The product mixture was obtained after the reaction was diluted with 360 μL of PBS buffer and then incubated with the as-prepared substrate at 37°C for 2 h. The substrate was washed with 2× SSC solution containing 0.1% (w/v) SDS for 5 min, washed with DI water several times, and dried before the Raman signal measurement.

### Control Experiments in SERS Measurements

The feasibility of our assays was verified by a series of control groups in the tests, including (A) C; (B) C + P; (C) C + P + M; (D) C + M + Exo III; (E) C + P + W + Exo III; and (F) C + P + M + Exo III. All control groups were performed in the identical conditions.

### Limit of Detection of the Assay

A series of ctDNA with gradient concentrations were prepared to determine the LOD of our assay. About 10 μL of P (10 μM) and 10 μL of ctDNA with different concentrations were mixed for incubation at 37°C for 2 h. The final concentrations of mutant ctDNA were in the range of 10–100 nM.

### Treatment of Blood Samples

The blood samples from healthy human donators were collected and treated according to the standard protocol. All human care and experimental procedures were conducted in compliance with relevant laws and the guidelines approved by the institutional committees for Human/Animal Experiments of the School of Basic Medical Sciences of Soochow University. An informed consent was acquired from all human subjects. The serum was obtained from the whole blood and mixed with H3.3-mutated ctDNA of various concentrations. Then, H3.3-mutated ctDNA was extracted from the serum according to the extraction procedure of QIAmp DNA Blood Mini Kit. Briefly, 100 μL of serum mixed with H3.3-mutated ctDNA was taken into a 1.5 mL centrifuge tube, then 10 μL of protease K and 100 μL of AL buffer were added, and later incubated at 56°C for 10 min. After adding 50 μL of ethanol, the solution was centrifuged for 1 min at 8,000 rpm. About 500 μL of AW1 and 500 μL of AW2 were added to the separation tube and centrifuged for 1 min at 8,000 rpm. After 100 μL of AE eluent was added, the solution was balanced for 1 min at room temperature and centrifuged for 1 min at 14,000 rpm to collect the eluent.

### Statistical Analysis

Statistical analyses were assessed with one-way ANOVA by using Prism 5.0 software (GraphPad Prism, United States). All intensities at the specified SERS peaks are presented as mean ± SD.

## Results and Discussion

### The Assay Principle of ctDNA Detection and Characterization of the Substrate

**FIGURE 1:**
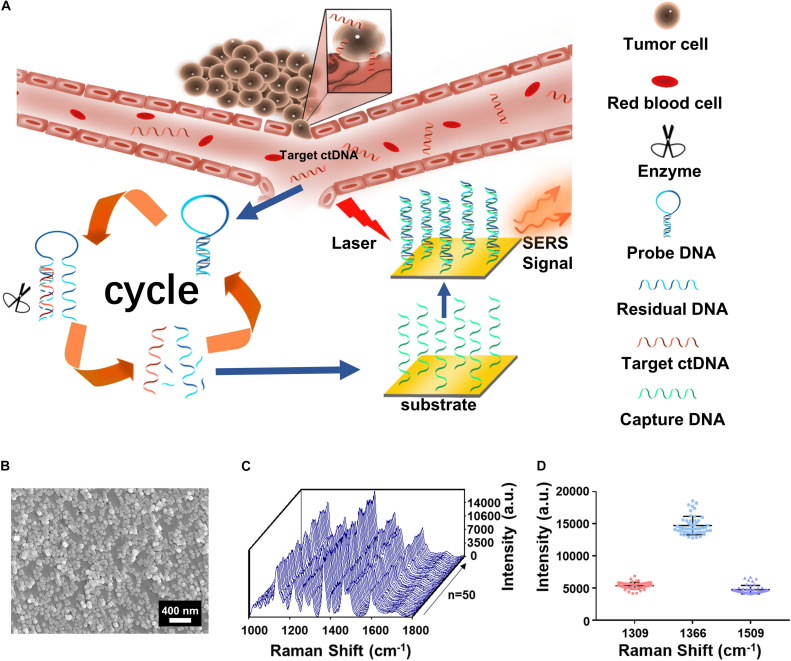
**(A)** The integration of cycled enzymatic DNA cleavage/amplification and surface-enhanced Raman scattering (SERS) for sensitive detection of ctDNA. **(B)** The scanning electron microscopy (SEM) image of the Au NPs@Si substrate. **(C)** SERS spectra of testing Cy5 collected from 50 random spots on the Au NPs@Si substrate in a single assay. **(D)** Averaged SERS intensities at 1,309, 1,366, and 1,509 cm^− 1^ peak from the 50 random spots, respectively (excitation wavelength: 633 nm, acquisition time: 10 s, laser power: 20 mW, the filter: d0.3).

The assay is featured with integration of the cycled enzymatic DNA cleavage/amplification and SERS for the sensitive detection of ctDNA (Figure 1A). An oligonucleotide probe is designed to be folded in a stem–loop hairpin structure, tagged with cyanine dye Cy5 at the 5’ end. The hairpin structure is stable, minimizing the undesired side hybridization with W sequence. In contrast, the stem–loop structure of the oligo probe can undergo changes to form a new double helix by hybridizing its 3’ end with the target sequence of the mutant ctDNA. Consequently, the protruding 3’ end in the new double helix can specifically be recognized by Exo III enzyme for the cleavage into nucleotides in a stepwise manner. Importantly, after completion of the cleavage process, the target sequence of ctDNA can be released into the solution and recycled for the next round of enzymatic DNA cleavage of the oligo probe. In this way, the residual DNA sequence generated by the digestion of the oligo probe can be accumulated to a great number by this cycled reaction. Hybridization of the amplified R and C preimmobilized on Au NPs@Si will bring the Cy5 tag close to the substrate, producing intensive SERS signals with a high efficiency. Therefore, a trace amount of ctDNAs can initiate the cycled generation of residual DNA sequences for amplified SERS detection.

Scanning electronic microscopy (SEM) (Figure 1B) suggested that the gold nanoparticles were uniform in size (60 nm) on the Au NP@Si substrate with a good distribution. The reproducibility in generating SERS signals was evaluated by scanning the substrate for spectral acquisition of up to 50 randomly selected spots. As shown in Figures 1C,D, there were minimal spot-to-spot variations of the intensities in the SERS spectra, with the coefficient variation values at the characteristic peaks of Cy5 at 1,309 cm^–1^ (CV < 9.88%), 1,366 cm^–1^ (CV < 9.73%), and 1,509 cm^–1^ (CV < 13.70%) and enhancement factor (EF) > 1.9 × 10^6^. The details of calculating the EF are listed in the Supplementary Information. We tested the SERS spectra at different laser powers by triturating the laser filters (Supplementary Figure 2A) and demonstrated the robustness of spectral data acquisition by switching laser power grades (high or medium) in five cycles (Supplementary Figure 2B). Therefore, the Au NP@Si substrate can serve as a reliable platform for SERS measurements of a trace amount of molecules.

### Oligo Sequence Optimization for ctDNA Detection

**FIGURE 2:**
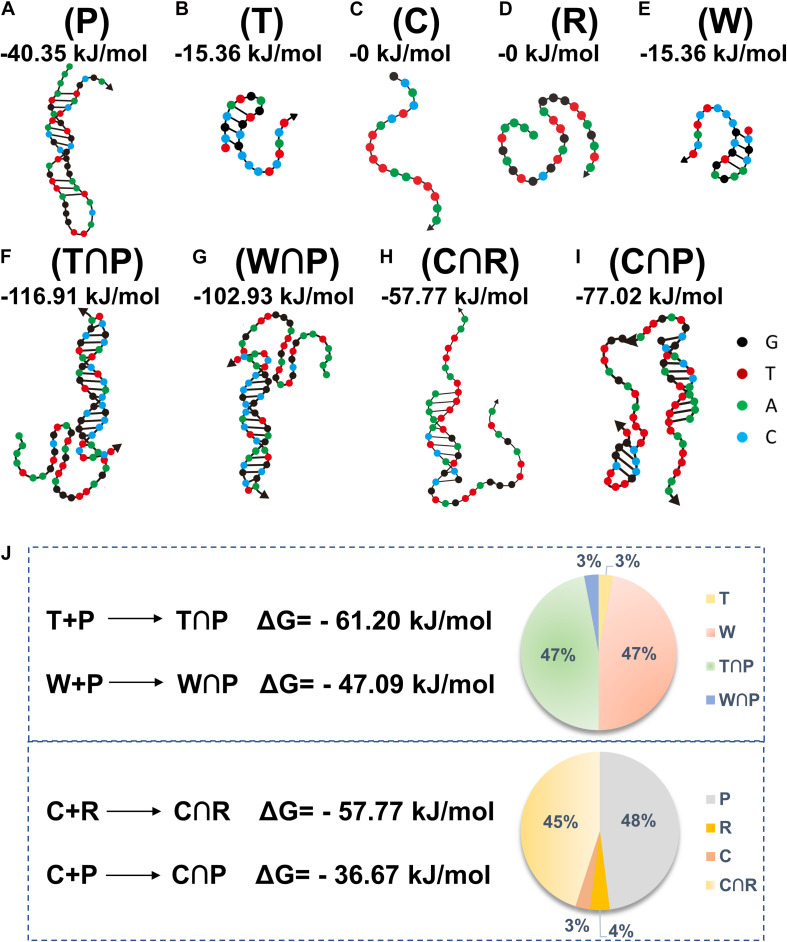
The structures of different oligos **(A–E)** (oligo P, T, C, R, and W) and oligo hybridization with annotation of free energy calculated by NUPACK **(F–I)** (T∩P, W∩P, C∩R, C∩P). Theoretical calculations of the energy changes for the competitive hybridization between DNA oligos at 37°C **(J)**.

The DNA sequences, including P, T, C, R, and W, are summarized in Table 1. Based on the literature reports (Schwartzentruber et al., 2012), the H3.3-mutated ctDNA from DIPG is different from W in the healthy samples by a single nucleotide mutation (A to T), as specified in Table 1. A theoretic analysis using the online software (NUPACK) was performed to determine the minimum free energy (MFE) secondary structures of these oligos and hybridization efficiencies between each other (Figure 2). P tended to keep hairpin structure in the solution with a relatively favorable free energy (−40.35 kJ mol^–1^) for the secondary structure (Figure 2A). The addition of H3.3-mutated ctDNA (T) to the solution would hybridize a segment of P competitively, thus changing its initial hairpin structure to form a partly hybridized helix with a 3’ blunt end. This hybridization was driven by the decrease of free energy (ΔG = −61.20 kJ mol^–1^). In addition, we compared the MFE changes for hybridization between W and P (ΔG = −47.09 kJ mol^–1^), which suggested that P would prefer to hybridize H3.3-mutated ctDNA, even if W had only one base change. We also calculated the occupancy rates of the various oligos by NUPACK, in order to investigate their hybridization efficiency in silica at 37 ^*o*^C. The calculation indicated that P was inclined to be hybridized with H3.3-mutated ctDNA with a high efficiency (nearly 47%); besides hybridization of 3% of W with P, the remaining W maintains its own structure (47%), leaving only 3% of the target DNA unhybridized in the solution (Figure 2J above, pie chart). We further compared the MFE changes for the hybridization scenarios between C and R (C∩R, ΔG = −57.77 kJ mol^–1^) or between C and P (C∩P, ΔG = −36.67 kJ mol^–1^). The results suggested that C would hybridize R, even when unreacted P was present in the solution. When the three kinds of DNA were mixed equally in mass, P would maintain its stable hairpin structure (48%), and most of the hybridization happened between C and R (45%, Figure 2J down, pie chart). This suggested that the presence of P (leftover after the cleavage reaction) would not influence the hybridization between R and C. Therefore, the oligo sequence design would promote the performance of our assay by minimizing the side reactions/hybridization of W∩P or C∩P as the sources of false-positive signals.

### Feasibility Tests of the Assay

**FIGURE 3:**
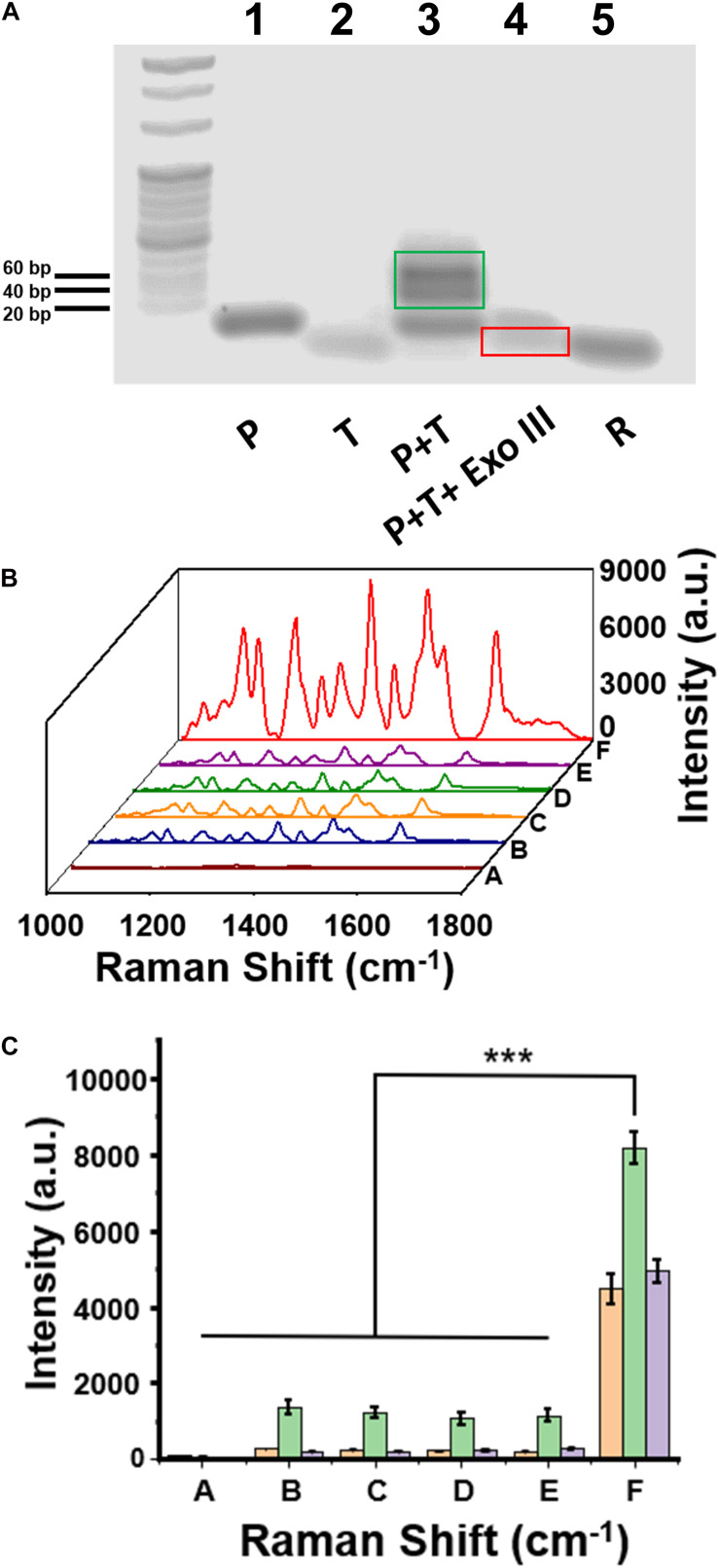
**(A)** The image of agarose gel electrophoresis to test the enzymatic reaction assisted by Exo III enzyme. **(B)** Surface-enhanced Raman scattering (SERS) spectra of a series of control samples on the capture DNA-Au NPs@Si substrate. **(C)** Intensity quantification of the SERS signals at the 1,309 cm, 1,366, and 1,509 cm^– 1^ peak, respectively. Sample (A) C, (B) C+P, (C) C+P+T, (D) C+T+Exo III, (E) C+P+wild-type DNA+Exo III, and (F) C+P+T+Exo III. Error bar: standard deviation (*n* = 3). The significant difference was calculated using one-way ANOVA, between the sample F and the negative controls (A–E) at the SERS peak of 1,366 cm^– 1^. ***P < 0.001 (excitation wavelength: 633 nm, acquisition time: 10 s, laser power: 20 mW, the filter: d0.3).

Agarose gel electrophoresis was employed to test DNA hybridization and Exo III enzyme–assisted DNA cleavage. As shown in Figure 3A, the bands in Lane 1, Lane 2, and Lane 5 were corresponding to P, H3.3-mutated ctDNA (T), and R in the gel electrophoresis, respectively. The hybridized product of T∩P was present as the dark band in Lane 3, with the light bands attributed to a small amount of the free oligos of P. After the addition of Exo III in Lane 4, the hybridized product of T∩P disappeared, suggesting an efficient DNA cleavage assisted by the Exo III enzyme. The residual DNA was observed after the enzymatic digestion, which highly contributed to the degradation of the hybridized product of T∩P by Exo III. GelRed usually stains double-stranded nucleic acids than single-stranded nucleic acids. It was interpreted that the band of T or R was weak because T tended to form only a short segment of double helix structure, while R was mostly single-stranded.

The feasibility of our assay was also validated with SERS measurements of a series of DNA oligo mixtures with or without Exo III enzyme, including five negative control groups and one positive experimental group (Figure 3B). There were no detectable SERS signals when only C was present on the substrate (curve A in Figure 3B); the group of C+P (curve B) and C+P+T (curve C) observed the same trend. These experiments suggested that C would not hybridize to P, which was consistent to our design and the theoretical calculation. We did not observe SERS signals for C+T+Exo III (curve D) or C+P+W+Exo III (curve E). In contrast, there was a significantly intensive SERS signal on the Au NPs@Si substrate in the positive experiment containing C, P, T, and Exo III (Curve F in Figure 3B). The signal intensity of the positive group (*F*) was nearly six to seven times higher than the other negative groups of *B*, *C*, *D*, and *E* at the characteristic peaks of Cy5 tag, such as 1,309, 1,366 cm, or 1,509 cm^–1^ (Figure 3C). The SERS signal comparison between E and F verified that the assay was highly selective to distinguish a single-nucleotide difference between W and M sequences. In addition, we performed the reaction (C+P+target DNA+Exo III) in two separate substrates in parallel to evaluate the substrate-to-substrate variation, which suggested reproducible SERS signal acquisition (Supplementary Figure 3).

### Specificity and Limit of Detection

**FIGURE 4:**
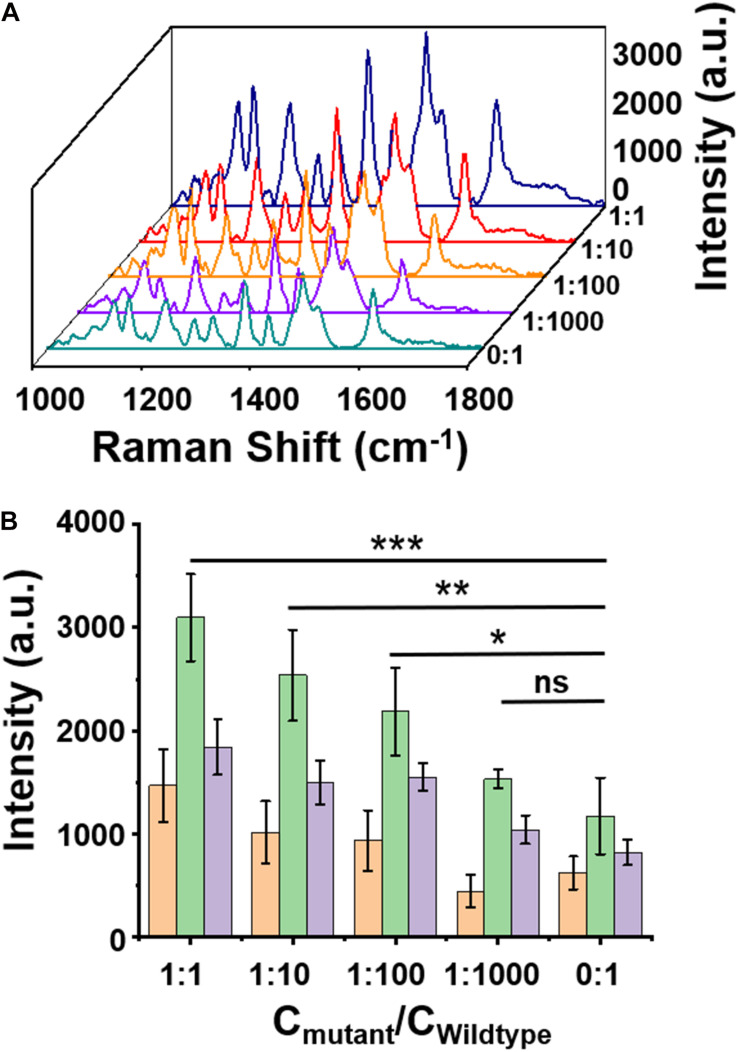
**(A)** A surface-enhanced Raman scattering (SERS) image of wild-type DNA with a concentration of 1 pM mixed with mutant DNA in different ratios. **(B)** Intensity quantification of the SERS signals at 1,309, 1,366, 1,509 cm^– 1^ peak, respectively. Error bar: standard deviation (*n* = 3). The significant difference was calculated using one-way ANOVA, between the individual titrated samples and the control (0:1) at the SERS peak of 1,366 cm^– 1^. ns: no significant difference, *P < 0.05, **P < 0.01, and ***P < 0.001 (excitation wavelength: 633 nm, acquisition time: 10 s, laser power: 20 mW, the filter: d0.3).

The specificity of the assay was further examined by mixing the W (1 pM) and H3.3-mutated ctDNA oligos in gradient concentrations at different ratios (1 pM, 0.1 pM, 0.01 pM, 1 fM, and 0 fM). As shown in Figure 4A, when the concentrations of H3.3-mutated ctDNA were reduced from 1 pM to 0 fM, the SERS signal intensities gradually decreased with the gradient changes, while maintaining the spectral patterns of Cy5 tag. Quantitative analysis of the characteristic peak intensities at 1,306 cm, 1,366 cm, and 1,509 cm^–1^ also demonstrated this trend (Figure 4B), suggesting that the assay can specifically distinguish the samples when the H3.3-mutated ctDNA oligos were diluted by 2 or 3 orders of magnitude with W.

**FIGURE 5:**
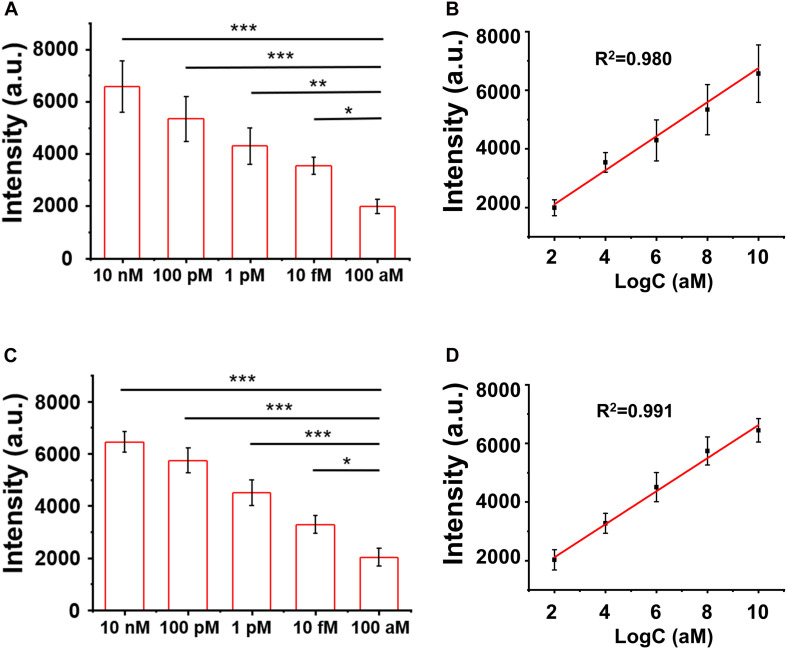
**(A,B)** Surface-enhanced Raman scattering (SERS) intensity and the linear fitting of signal-to-noise ratios at the SERS peak of 1,366 cm^– 1^ with circulating tumor DNA in phosphate buffer solution (PBS). **(C,D)** SERS spectra and the linear fitting of signal-to-noise ratios at the SERS peak of 1,366 cm^– 1^ with ctDNA in the blood. Error bar: standard deviation (*n* = 3). The significant difference was calculated using one-way ANOVA, between the individual titrated samples against the most diluted one (100 nM) at the SERS peak of 1366 cm^–1^. *P < 0.05, **P < 0.01, and ***P < 0.001 (excitation wavelength: 633 nm, acquisition time: 10 s, laser power: 20 mW, the filter: d0.3).

The LOD of the assay was evaluated by titrating the H3.3-mutated ctDNA oligo concentrations in the PBS buffer and the human serum, separately. As shown in Figures 5A,B, the SERS signal intensities of the characteristic peak at 1,366 cm^–1^ decreased when the ctDNA oligo concentration was diluted from 10 to 100 nM in PBS buffer. There was a good linear relationship (*R*^2^ = 0.980) between the SERS signal intensities and ctDNA oligo concentrations, with an LOD of 7.9 fM (1.0 pg mL^–1^) (signal-to-noise ratio ≥ 3).

Furthermore, we performed a pilot experiment to test the assay in human serum samples. H3.3-mutated ctDNA oligos were spiked into the human serum samples from healthy donors with a concentration gradient from 10 to 100 nM. The DNA samples were then extracted from the mixture by referring to a standard DNA extraction procedure, in order to remove serum proteins or other contamination sources. As shown in Figures 5C,D, we observed a strong correlation (linearity *R*^2^ = 0.991) between the SERS signal intensities and the spiked concentrations of the H3.3-mutated DNA oligos. The LOD was estimated to be 9.1 fM (1.2 pg mL^–1^) according the criterion of the signal-to-noise ratio ≥3. The experiments suggested that the SERS/enzyme amplification technique allowed for sensitive detection of M in complicated samples, thus offering a useful tool for potential clinical translation.

Diffuse intrinsic pontine gliomas is a brainstem tumor of malignancy in childhood for which median survival is below 1 year (Hoffman et al., 2018). More than 90% of patients with DIPG are estimated to have the point mutation in H3.3 (65% of tumors) or H3.1 (25% of tumors), while the rest are estimated to have histone 3 wild-type tumors (Vanan and Eisenstat, 2015). Among those mutations, the averaged survival span of H3.3 mutation is the shortest (10.4 months) (Cordero et al., 2017), thus being in an urgent need of developing sensitive diagnostic tools. By using the spiked blood samples, we have demonstrated an ultra-sensitive detection of the target DNA sequence for H3.3 mutation of DIPG with an LOD of 9.1 fM (1.2 pg ml^–1^), nearly 10–100 folds lower than the typical ctDNA concentration range (0.01–0.1 ng ml^–1^) of the disease. This study has not tested the blood samples from patients with DIPG yet due to a limitation of the clinical resources. It is necessary to further validate this technique in carefully grouped patient blood samples with references to the clinical outcomes in the future.

In our technique, the design of P is very flexible for the introduction of any target DNA sequence, thus allowing for an easy translation to sensitive detection of the ctDNA of other types of cancers without changing the enzyme. For instance, researchers have identified a variety of clinically useful mutations in the ctDNA samples, including G protein subunit alpha Q (GNAQ) Q209L mutation (626A > T) in metastatic uveal melanoma (Madic et al., 2012), p110a catalytic subunit of the class 1A PI3K (PIK3CA) E545K mutation (1633G > A) in breast cancer (Board et al., 2010), or epidermal growth factor receptor (EGFR) T790M mutation (2369C > T) in non-small-cell (Normanno et al., 2017). The applications of the technique on the sensitive detection of these mutations are promising in facilitating the early diagnosis of the diseases and treatment with personalized medicine. The key for successful development of a new assay by the technique is careful optimization of the competitive hybridization segment of P for minimal side reactions of hybridizing W or C.

## Conclusion

In summary, we have developed a method that integrates cycled enzymatic DNA amplification and SERS spectroscopy for sensitive detection of ctDNA, by combining the advantages of highly efficient amplification by the Exo III enzyme and the SERS technique. It brings the LOD to as low as 7.9 fM in the mixture solution or 9.1 fM in the spiked serum samples. Our approach can differentiate a single-base mismatch between wide-type DNAs and mutated DNAs, which has better applications in medical laboratory research and translation for early clinical diagnosis.

## Data Availability Statement

The raw data supporting the conclusions of this article will be made available by the authors, without undue reservation.

## Ethics Statement

The studies involving human participants were reviewed and approved by the institutional committees for Human/Animal Experiments of the School of Basic Medical Sciences of Soochow University. The patients/participants provided their written informed consent to participate in this study.

## Author Contributions

JL and XM conceived and designed the experiments. XM and QF performed the experiments. XX and SL provided help with data analysis. RW assisted the characterization of the Au NP@Si substrates. JY provided help in the gel electrophoresis experiments. XM, BN, and JL wrote the manuscript. All authors contributed to the article and approved the submitted version.

## Conflict of Interest

The authors declare that the research was conducted in the absence of any commercial or financial relationships that could be construed as a potential conflict of interest.

## References

[B1] Alix-Panabieres C. Pantel K. (2016). Clinical applications of circulating tumor cells and circulating tumor dna as liquid biopsy. *Cancer Discov.* 6 479–491. 10.1158/2159-8290.CD-15-1483 26969689

[B2] Bettegowda C. Sausen M. Leary R. J. Kinde I. Wang Y. X. Agrawal N. (2014). Detection of circulating tumor DNA in Early- and late-stage human malignancies. *Sci. Transl. Med.* 6:224ra224. 10.1126/scitranslmed.3007094 24553385 PMC4017867

[B3] Board R. E. Wardley A. M. Dixon J. M. Armstrong A. C. Howell S. Renshaw L. (2010). Detection of PIK3CA mutations in circulating free DNA in patients with breast cancer. *Breast Cancer Res. Treat.* 120 461–467. 10.1007/s10549-010-0747 20107891

[B4] Chen Y. Liu H. Tian Y. Du Y. Ma Y. Zeng S. (2020). In situ recyclable surface-enhanced Raman scattering-based detection of multicomponent pesticide residues on fruits and vegetables by the flower-like MoS2@ Ag hybrid substrate. *ACS Appl. Mater. Interf.* 12 14386–14399. 10.1021/acsami.9b22725 32118398

[B5] Cordero F. J. Huang Z. Grenier C. He X. Hu G. McLendon R. E. (2017). Histone H3. 3K27M represses p16 to accelerate gliomagenesis in a murine model of DIPG. *Mol. Cancer Res.* 15 1243–1254. 10.1158/1541-7786 28522693 PMC5581686

[B6] Dawson S. J. Tsui D. W. Y. Murtaza M. Biggs H. Rueda O. M. Chin S. F. (2013). Analysis of circulating tumor DNA to monitor metastatic breast cancer. *N. Engl. J. Med.* 368 1199–1209. 10.1056/NEJMoa1213261 23484797

[B7] Diaz L. A. Bardelli A. (2014). Liquid biopsies: genotyping circulating tumor DNA. *J. Clin. Oncol.* 32 579–579. 10.1200/JCO.2012.45.2011 24449238 PMC4820760

[B8] Diehl F. Schmidt K. Choti M. A. Romans K. Goodman S. Li M. (2008). Circulating mutant DNA to assess tumor dynamics. *Nat. Med.* 14 985–990. 10.1038/nm.1789 18670422 PMC2820391

[B9] Drummond T. G. Hill M. G. Barton J. K. (2003). Electrochemical DNA sensors. *Nat. Biotechnol.* 21 1192–1199. 10.1038/nbt873 14520405

[B10] Du Y. Liu H. Chen Y. Tian Y. Zhang X. Gu C. (2020). Recyclable label-free SERS-based immunoassay of PSA in human serum mediated by enhanced photocatalysis arising from Ag nanoparticles and external magnetic field. *Appl. Surf. Sci.* 528 146953–146953. 10.1016/j.apsusc.2020.146953

[B11] Fan M. Andrade G. F. S. Brolo A. G. (2020). A review on recent advances in the applications of surface-enhanced Raman scattering in analytical chemistry. *Anal. Chim. Acta* 1097 1–29. 10.1016/j.aca.2019.11.049 31910948

[B12] Forshew T. Murtaza M. Parkinson C. Gale D. Tsui D. W. Kaper F. (2012). Noninvasive identification and monitoring of cancer mutations by targeted deep sequencing of plasma DNA. *Sci. Transl. Med.* 4:136ra168. 10.1126/scitranslmed.3003726 22649089

[B13] Hoffman L. M. Van Zanten S. E. V. Colditz N. Baugh J. Chaney B. Hoffmann M. (2018). Clinical, radiologic, pathologic, and molecular characteristics of long-term survivors of diffuse intrinsic pontine glioma (DIPG): a collaborative report from the International and European society for pediatric oncology DIPG registries. *J. Clin. Oncol.* 36 1963–1963. 10.1200/JCO.2017.75.9308 29746225 PMC6075859

[B14] Janne P. A. Borras A. M. Kuang Y. Rogers A. M. Joshi V. A. Liyanage H. (2006). A rapid and sensitive enzymatic method for epidermal growth factor receptor mutation screening. *Clin. Cancer Res.* 12(3 Pt 1), 751–758. 10.1158/1078-0432.CCR-05-2047 16467085

[B15] Kitchin P. A. Szotyori Z. Fromholc C. Almond N. (1990). Avoidance of false positives. *Nature* 344 201–201. 10.1038/344201a0 2156164

[B16] Lai J. Du B. Wang Y. Wu R. Yu Z. (2018). Next-generation sequencing of circulating tumor DNA for detection of gene mutations in lung cancer: implications for precision treatment. *Oncol. Targets Ther.* 11 9111–9116. 10.2147/OTT.S174877 30588023 PMC6299472

[B17] Langer J. Jimenez de Aberasturi D. Aizpurua J. Alvarez-Puebla R. A. Auguie B. Baumberg J. J. (2020). Present and future of surface-enhanced raman scattering. *ACS Nano* 14 28–117. 10.1021/acsnano.9b04224 31478375 PMC6990571

[B18] Li M. Cushing S. K. Zhou G. W. Wu N. Q. (2020). Molecular hot spots in surface-enhanced Raman scattering. *Nanoscale* 12 22036–22041. 10.1039/D0NR06579J 33146197

[B19] Li M. Diehl F. Dressman D. Vogelstein B. Kinzler K. W. (2006). BEAMing up for detection and quantification of rare sequence variants. *Nat. Methods* 3 95–97. 10.1038/NMETH850 16432518

[B20] Li N. Hao X. Kang B. H. Xu Z. Shi Y. Li N. B. (2016). Enzyme-free fluorescent biosensor for the detection of DNA based on core-shell Fe3O4 polydopamine nanoparticles and hybridization chain reaction amplification. *Biosens. Bioelectron.* 77 525–529. 10.1016/j.bios.2015.10.004 26469729

[B21] Li R. Zou L. Luo Y. Zhang M. Ling L. (2017). Ultrasensitive colorimetric detection of circulating tumor DNA using hybridization chain reaction and the pivot of triplex DNA. *Sci. Rep.* 7 44212–44212. 10.1038/srep44212 28276503 PMC5343571

[B22] Liang Z. Zhou J. Petti L. Shao L. Jiang T. Qing Y. (2019). SERS-based cascade amplification bioassay protocol of miRNA-21 by using sandwich structure with biotin–streptavidin system. *Analyst* 144 1741–1750. 10.1039/c8an02259c 30663745

[B23] Lin D. Wu Q. Qiu S. Chen G. Feng S. Chen R. (2019). Label-free liquid biopsy based on blood circulating DNA detection using SERS-based nanotechnology for nasopharyngeal cancer screening. *Nanomedicine* 22 102100–102100. 10.1016/j.nano.2019.102100 31648038

[B24] Madic J. Piperno-Neumann S. Servois V. Rampanou A. Milder M. Trouiller B. (2012). Pyrophosphorolysis-activated polymerization detects circulating tumor DNA in metastatic uveal melanoma. *Clin. Cancer Res.* 18 3934–3941. 10.1158/1078-0432.CCR-12-0309 22645051

[B25] Mathai S. S. Adhikari K. M. (2013). Repeated false positive HIV DNA PCR in an exposed infant. *Med. J. Armed. Forc. India* 69 392–393. 10.1016/j.mjafi.2013.07.004 24600150 PMC3862937

[B26] Newton C. R. Graham A. Heptinstall L. E. Powell S. J. Summers C. Kalsheker N. (1989). Analysis of any point mutation in DNA - the Amplification Refractory Mutation System (Arms). *Nucleic Acids Res.* 17 2503–2516. 10.1093/nar/17.7.2503 2785681 PMC317639

[B27] Normanno N. Denis M. G. Thress K. S. Ratcliffe M. Reck M. (2017). Guide to detecting epidermal growth factor receptor (EGFR) mutations in ctDNA of patients with advanced non-small-cell lung cancer. *Oncotarget* 8:12501. 10.18632/oncotarget.13915 27980215 PMC5355360

[B28] Reinert T. Henriksen T. V. Rasmussen M. H. Sethi H. Sharma S. Wu H. T. (2018). Personalized circulating tumor DNA analysis to monitor colorectal cancer. *Cancer Res.* 78 1590–1590. 10.1158/1538-7445

[B29] Schwartzentruber J. Korshunov A. Liu X. Y. Jones D. T. Pfaff E. Jacob K. (2012). Driver mutations in histone H3.3 and chromatin remodelling genes in paediatric glioblastoma. *Nature* 482 226–231. 10.1038/nature10833 22286061

[B30] Szekeres G. P. Kneipp J. (2019). SERS probing of proteins in gold nanoparticle agglomerates. *Front. Chem.* 7:30. 10.3389/fchem.2019.00030 30766868 PMC6365451

[B31] Taly V. Pekin D. Benhaim L. Kotsopoulos S. K. Le Corre D. Li X. Y. (2013). Multiplex picodroplet digital PCR to detect KRAS mutations in circulating DNA from the plasma of colorectal cancer patients. *Clin. Chem.* 59 1722–1731. 10.1373/clinchem.2013.206359 23938455

[B32] Vanan M. I. Eisenstat D. D. (2015). DIPG in children–what can we learn from the past? *Front. Oncol.* 5:237. 10.3389/fonc.2015.00237 26557503 PMC4617108

[B33] Vogelstein B. Kinzler K. W. (1999). Digital PCR. *Proc. Natl. Acad. Sci. U.S.A.* 96 9236–9241. 10.1073/pnas.96.16.9236 10430926 PMC17763

[B34] Wan J. C. M. Massie C. Garcia-Corbacho J. Mouliere F. Brenton J. D. Caldas C. (2017). Liquid biopsies come of age: towards implementation of circulating tumour DNA. *Nat. Rev. Cancer* 17 223–238. 10.1038/nrc.2017.7 28233803

[B35] Wee E. J. Wang Y. Tsao S. C. Trau M. (2016). Simple, sensitive and accurate multiplex detection of clinically important melanoma DNA mutations in circulating tumour DNA with SERS nanotags. *Theranostics* 6 1506–1513. 10.7150/thno.15871 27446486 PMC4955051

[B36] Weiss L. Hufnagl C. Greil R. (2013). Circulating tumor DNA to monitor metastatic breast cancer. *N. Engl. J. Med.* 369 93–93. 10.1056/NEJMc1306040 23822790

[B37] Yang H. Gao Y. Wang S. Qin Y. Xu L. Jin D. (2016). In situ hybridization chain reaction mediated ultrasensitive enzyme-free and conjugation-free electrochemcial genosensor for BRCA-1 gene in complex matrices. *Biosens. Bioelectron.* 80 450–455. 10.1016/j.bios.2016.02.011 26875018

