# Supplementary material for: Integrating Cycled Enzymatic DNA Amplification and Surface-Enhanced Raman Scattering for Sensitive Detection of Circulating Tumor DNA
Source: Front Mol Biosci. 2021 May 4;8:676065. doi: 10.3389/fmolb.2021.676065 (PMC8129026; doi:10.3389/fmolb.2021.676065)
Supplement: Supplementary file 1 [file Table_1.DOCX]

**Supporting information**

**Integrating Cycled Enzymatic DNA Amplification and Surface-Enhanced Raman Scattering for Sensitive Detection of Circulating Tumor DNA**

Xinxing Miao^†1^, Qianqian Fang^†1^, Xiang Xiao^1^, Sidi Liu^1^, Renfei Wu^1^, Jun Yan^1^, Baoqing Nie^*2^, Jian Liu^*1^

^1^ Institute of Functional Nano and Soft Materials (FUNSOM), Jiangsu Key Laboratory for Carbon-Based Functional Materials and Devices, Soochow University, Suzhou, Jiangsu Province 215123, China

^2^ School of Electronic and Information Engineering, Soochow University, Suzhou, Jiangsu Province 215123, China

The Enhancement Factor (EF) of the substrate is calculated according to the following equation:

$$EF=\frac{I_{SERS}\times N_{bulk}}{I_{bulk}\times N_{SERS}}=\frac{I_{SERS}}{I_{bulk}}\times\frac{S_{SERS}\times V_{bulk}\times C_{bulk}}{S_{bulk}\times V_{SERS}\times C_{SERS}}$$

In our SERS measurements, 10 μl of 1 μM Cy5 aqueous solution was dispersed on the Au NPs@Si substrate, and then 10 μl of 10 mM Cy5 solution was dispersed on the clean silicon wafer. The Raman intensities at 1366 cm^-1^ were measured for these two different types of substrates. Separate SERS measurements were performed on 50 randomly-selected locations of each substrate to determine the averaged intensity values. Based on the results of the measurements, the averaged EF value was determined to be 1.9×10^6^ for the AuNPs@Si substrate.

Table S1 Correlation of the characteristic Raman bands of Cy5 and the chemical bonds ^1-3^.

| The Raman bands of Cy5 (cm^-1^) | Assignments |
| --- | --- |
| 1230 | **C-N stretching** |
| 1309 | **δ(COH)/δ(COH)/δ(COH)/ν(CC)** |
| 1366 | **δ(COH)/ν(CC)** |
| 1509 | **C=C ring-stretching** |
| 1605 | **C=N stretching** |


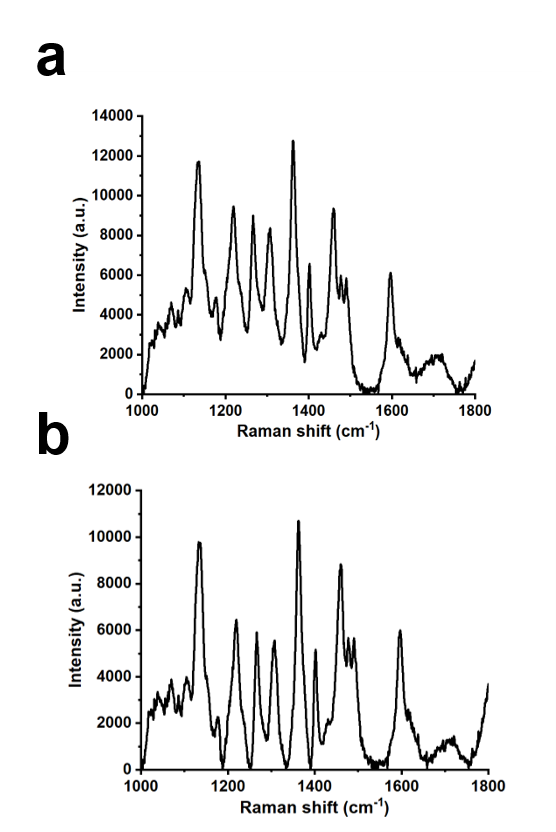


Figure S1. SERS spectra of before (a) and after (b) removing the background baseline. (excitation wavelength: 633 nm, acquisition time: 10 sec, laser power: 20 mW, the filter: d0.3)


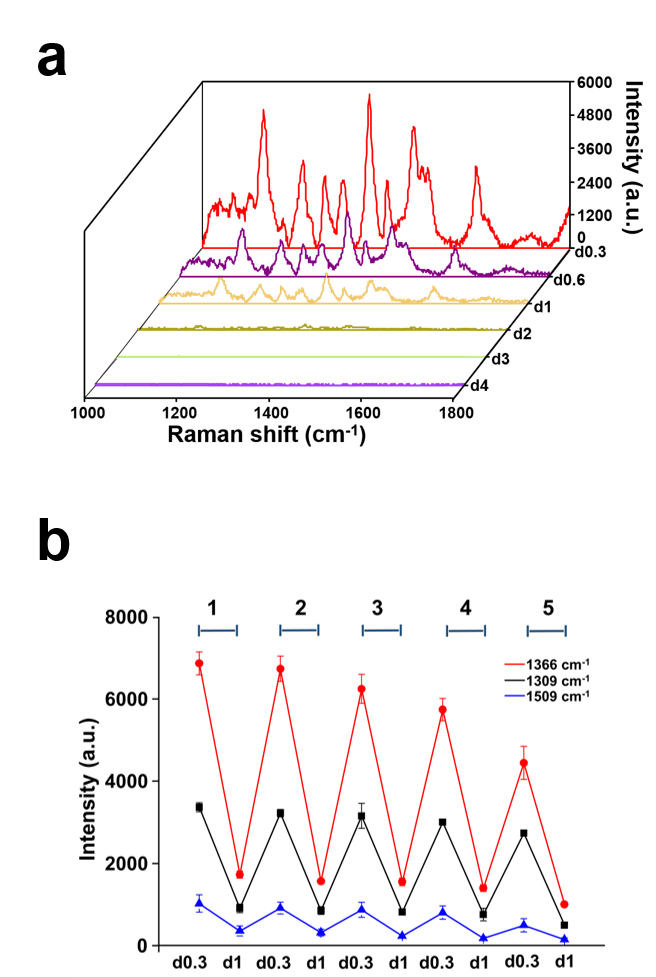


Figure S2. (a) The SERS spectra at different laser powers by triturating the laser filters, including d0.3, d0.6, d1, d2, d3, d4 and the Raman signal to repeat the procedure of irradiation with the high laser power (d0.3) and medium laser power (d1) in five cycles (b). (excitation wavelength: 633 nm, acquisition time: 10 sec, laser power: 20 mW)


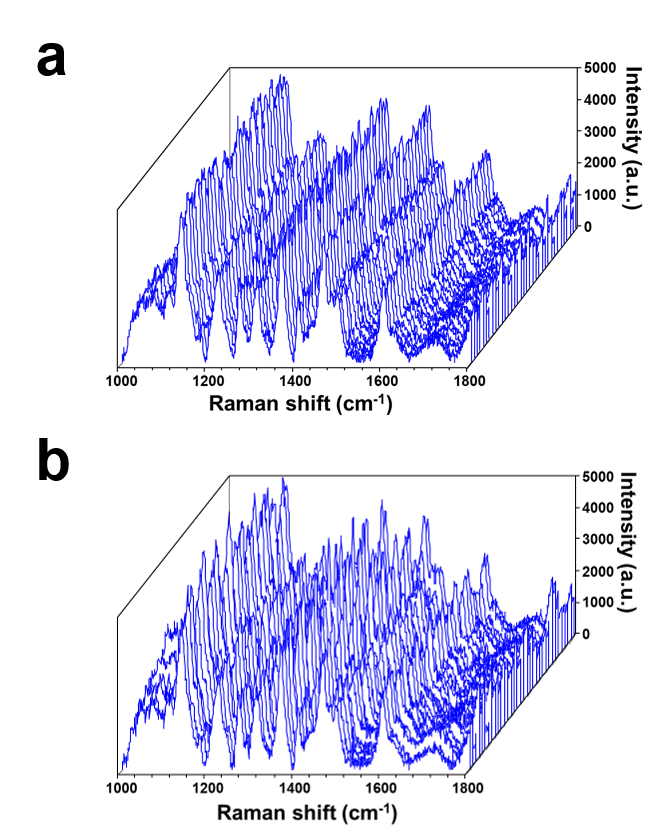


Figure S3. The SERS spectra of the reaction (C+P+target DNA+Exo III) in two separate substrates in parallel. (excitation wavelength: 633 nm, acquisition time: 10 sec, laser power: 20 mW, the filter: d0.3)

1. Zhan, P.; Wen, T.; Wang, Z. g.; He, Y.; Shi, J.; Wang, T.; Liu, X.; Lu, G.; Ding, B., DNA Origami Directed Assembly of Gold Bowtie Nanoantennas for Single‐Molecule Surface‐Enhanced Raman Scattering. *Angewandte Chemie International Edition* **2018,** *57* (11), 2846-2850.

2. Novara, C.; Petracca, F.; Virga, A.; Rivolo, P.; Ferrero, S.; Chiolerio, A.; Geobaldo, F.; Porro, S.; Giorgis, F., SERS active silver nanoparticles synthesized by inkjet printing on mesoporous silicon. *Nanoscale Res Lett* **2014,** *9* (1), 1-7.

3. Xu, L.; Zhao, S.; Ma, W.; Wu, X.; Li, S.; Kuang, H.; Wang, L.; Xu, C., Multigaps embedded nanoassemblies enhance in situ Raman spectroscopy for intracellular telomerase activity sensing. *Adv Funct Mater* **2016,** *26* (10), 1602-1608.
